# Short-term Antihyperglycemic and Weight Modulation Effects of *Leuenbergeria bleo* (Kunth) Lodé: Phytochemical Screening and in vivo Assessment in an Alloxan-Induced Diabetic Rat Model

**DOI:** 10.5812/ijpr-164873

**Published:** 2025-12-14

**Authors:** Sheryar Afzal, Velaga Venkata Sathya Sai Appalaraju, Ali Attiq, Iram Malik, Ibrahim Albokhadaim, Sameer M Alhojaily, Yuan Seng Wu, Yasir A Almofty, Ahmed Omer Alameen, Ghazi Aljabl

**Affiliations:** 1 Department of Biosciences, College of Veterinary Medicine, King Faisal University, Hofuf, Saudi Arabia; 2 Department of Medicinal Chemistry, Faculty of Pharmacy and Biomedical Sciences, MAHSA University, Petaling Jaya, Malaysia; 3 School of Pharmaceutical Sciences, Universiti Sains Malaysia, George Town, Malaysia; 4 Department of Chemistry, Govt. Graduate College for Women, University of Sargodha, Sargodha, Pakistan; 5 Department of Biological Sciences, School of Medical and Life Sciences, Sunway University, Petaling Jaya, Malaysia

**Keywords:** *Leuenbergeria bleo*, Diabetes Mellitus, Phytochemicals, Alloxan, Antihyperglycemic Activity

## Abstract

**Background:**

*Leuenbergeria bleo*, a medicinal plant with traditional therapeutic uses, has attracted interest for its antidiabetic potential.

**Objectives:**

This study evaluated the antihyperglycemic effects of its leaf extracts in an alloxan-induced diabetic rat model.

**Methods:**

The leaves of *L. bleo* were sequentially extracted using petroleum ether, chloroform, and methanol. Phytochemical screening was conducted to identify bioactive compounds such as phytosterols, alkaloids, tannins, and flavonoids. Thirty healthy Albino Wistar rats were divided into five groups. Diabetes was induced in four groups via intraperitoneal injection of alloxan monohydrate (140 mg/kg). The diabetic rats were treated with either chloroform or methanol extracts of *L. bleo* (500 mg/kg/day) for five days, with metformin (100 mg/kg/day) serving as the standard reference treatment. Blood glucose levels were monitored daily, and body weight was recorded on days 1, 3, and 5.

**Results:**

Methanol extract significantly reduced blood glucose levels by 12.23% by day 5, compared to a 6.35% reduction with chloroform extract. Additionally, the methanol extracts mitigated weight loss, resulting in an 8.07% increase in body weight, while the diabetic control group experienced a 26.72% decrease. The effects of the methanol extract were comparable to metformin, though slightly less potent. *Leuenbergeria bleo* exhibits modest antihyperglycemic potential, particularly in its methanol extract, which also stabilizes body weight in diabetic rats.

**Conclusions:**

The presence of flavonoids and alkaloids likely contributes to these effects. Further research is warranted to elucidate the exact mechanisms and explore *L. bleo*’s potential as a natural alternative therapy for diabetes management.

## 1. Background

Diabetes mellitus is a chronic metabolic disorder characterized by persistent hyperglycemia due to insufficient insulin production or impaired cellular response (1, 2). It is classified into type I, type II, and gestational diabetes, with type II accounting for 90 - 95% of cases worldwide (3, 4). Lifestyle factors such as obesity and inactivity contribute significantly. In 2021, 537 million adults had diabetes globally, projected to reach 783 million by 2045 (5, 6). Although various antidiabetic drugs exist, many have limitations in long-term efficacy and cause adverse effects, fueling interest in safer, plant-based alternatives.

*Leuenbergeria bleo* (Kunth) Lode, known locally as "Jarum Tujuh Bilah" and "Cak Sing Cam", is a medicinal plant from the Cactaceae family, with approximately 8 accepted species and multiple synonyms (7-9). The species *L. bleo* (Kunth) Lode, formerly recognized as *Pereskia bleo* (Kunth) DC., has undergone taxonomic revision based on molecular phylogenetic evidence. Studies by Butterworth and Wallace and Nyffeler and Eggli revealed that the genus *Pereskia*, as traditionally defined, was paraphyletic, comprising distinct northern and southern evolutionary lineages. In response to these findings, Lode proposed the establishment of the genus *Leuenbergeria*, reclassifying *P. bleo* into this group, which is distinguished by its persistent foliage and woody shrub or tree morphology at the base of the Cactaceae family. Leading botanical databases such as Plants of the World Online (POWO 2024) and World Flora Online now recognize *L. bleo* as the valid name, with *P. bleo* listed as a synonym. This updated nomenclature reflects the current understanding of evolutionary relationships within the cactus family and is recommended for use in contemporary scientific literature (10-14). Native to regions from Brazil to Mexico, it is widely cultivated for its medicinal properties (9). Traditionally, *L. bleo* is used for detoxification, cancer, hypertension, diabetes, gastrointestinal disorders, pain, and inflammatory diseases (9, 15). Scientific studies have examined its anticancer, antihypertensive, antimicrobial, antidiabetic, and analgesic effects (16-20), but conclusive evidence is lacking, especially regarding its antidiabetic activity.

## 2. Objectives

This study aims to address this gap by evaluating the antihyperglycemic effects of *L. bleo* extracts in an alloxan-induced diabetic rat model. It is hypothesized that antioxidant properties of the extracts protect pancreatic β-cells from oxidative stress. The research also includes screening for active constituents using chromatographic and spectroscopic techniques. The findings could support the development of novel, cost-effective, and safer herbal formulations for diabetes management, providing scientific validation for traditional uses and informing new natural therapeutic approaches amid the global diabetes rise.

## 3. Methods

### 3.1. Plant Material Collection

*Leuenbergeria bleo* leaves were collected in July 2019 from Bukit Mertajam, Penang, and authenticated by Mr. Kamarudin Saleh, botanist at Forest Research Institute Malaysia (FRIM). A voucher specimen (PID 220914-17) was deposited at the FRIM herbarium. Leaves were cleaned, shade-dried, ground to powder, and stored for extraction.

### 3.2. Preparation of Plant Extracts

Approximately 300 g of powdered leaves were sequentially extracted using petroleum ether, chloroform, and methanol via Soxhlet apparatus. Residual plant material was dried for 48 hours before the next solvent extraction. Extracts were dried with a rotary evaporator at 40°C, yielding petroleum ether (16.79 g), chloroform (15.67 g), and methanol (17.81 g) extracts, which were stored at 4°C until analysis.

### 3.3. Preliminary Phytochemical Analysis

Qualitative screening for alkaloids, flavonoids, glycosides, tannins, phenolics, and phytosterols was performed using previously reported methods with slight modifications (Table 1) (17).

**Table 1.** Qualitative Phytochemical Tests Used for the Screening of *Leuenbergeria bleo* Extracts

Phytochemical Class Test	Procedure	Positive Result
**Carbohydrates and glycosides**		
Molisch's test	A small quantity of the extract was dissolved in 4 mL of distilled water, followed by filtration. The filtrate was treated with 2 - 3 drops of 1% alcoholic α-naphthol and 2 mL of concentrated sulfuric acid.	The formation of a brown ring at the junction of the two liquids
**Proteins and free amino acids**		
Million's test	The extract was dissolved in water, followed by the addition of Million's reagent.	The appearance of a red color
**Tannins and phenolic compounds**		
Ferric chloride test	2 mL of the extract was treated with 1 mL of 5% ferric chloride solution.	A blue or green coloration
**Phytosterols**		
Salkowski's test	5 mL of chloroform was added to the extract, followed by a few drops of concentrated sulfuric acid.	The appearance of a brown color
Libermann-Burchard's test	The extract was dissolved in chloroform, treated with concentrated sulfuric acid and acetic anhydride.	The appearance of a bluish-green color
**Alkaloids**		
Wagner's	The extract was treated with dilute hydrochloric acid and Wagner's reagent.	The formation of a reddish-brown precipitate
Hager's reagents	The extract was treated with dilute hydrochloric acid and Hager's reagent.	Yellow precipitate
**Flavonoids**		
Shinoda's test	The extract was dissolved in alcohol, and a small amount of magnesium and concentrated hydrochloric acid was added.	Magenta color

### 3.4. Experimental Animals

Thirty healthy Albino Wistar rats (8 - 10 weeks, 180 - 200 g) of either sex were acclimatized for one week under standard conditions (22 ± 2°C, 55 ± 5% humidity, 12 h light/dark cycle) with free access to food and water. Ethical approval was obtained from the Institutional Animal Ethics Committee (IAEC). All procedures involving animals were conducted in accordance with the ARRIVE guidelines and institutional animal welfare protocols. Efforts were made to minimize animal suffering, and the number of animals used was kept to the minimum required to achieve statistical validity.

### 3.5. Induction of Diabetes

Diabetes was induced via a single intraperitoneal injection of alloxan monohydrate (140 mg/kg) dissolved in saline after 18 h fasting. To prevent fatal hypoglycemia, 5% glucose solution was given for 24 h post-injection. Blood glucose was measured from the tail vein at 1, 12, 24, and 48 h. Rats with fasting glucose > 200 mg/dL at 48 h were considered diabetic. Six healthy rats served as nondiabetic controls.

### 3.6. Experimental Design

Diabetic rats were divided into five groups (n = 6 each): Animals were randomly assigned to treatment groups using a computer-generated randomization sequence. Blinding was implemented during outcome assessment, with investigators unaware of group allocation while recording blood glucose and body weight data. These procedures were followed to minimize bias and enhance reproducibility.

Group A (normal control, distilled water orally), group B (diabetic control, distilled water orally), group C (metformin 100 mg/kg/day intraperitoneally), group D (chloroform extract 500 mg/kg/day orally), group E (methanol extract 500 mg/kg/day orally). Treatments lasted 5 days with doses adjusted by body weight.

### 3.7. Blood Glucose Measurement

Blood glucose was measured daily from days 1 to 5 under fasting conditions via glucometer (Accu-Check Active, Roche Diagnostics, Germany) using tail vein samples at consistent times.

### 3.8. Body Weight Monitoring

Body weight was recorded on days 1, 3, and 5 using a digital balance to evaluate treatment effects.

### 3.9. Statistical Analysis

Data are expressed as mean ± SEM. Statistical significance was assessed by one-way ANOVA with Tukey’s post-hoc test using SPSS v19.0; P < 0.05 was considered significant.

## 4. Results

### 4.1. Extraction and Qualitative Phytochemical Analysis of Leuenbergeria bleo Leaves

The extraction of *L. bleo* leaves using solvents of increasing polarity — petroleum ether, chloroform, and methanol — yielded varying amounts of extract, indicating differential solubilities of the plant's bioactive compounds. Methanol, the most polar solvent used, produced the highest yield at 5.94%, followed by chloroform at 5.60% and petroleum ether at 5.22%. Phytochemical analysis of the *L. bleo* extracts revealed the presence of several key secondary metabolites, including steroids, alkaloids, tannins, phenolic compounds, and flavonoids. The presence of these bioactive compounds, particularly in the chloroform and methanol extracts, suggests that these solvents are more effective at isolating compounds with potential pharmacological activities (Table 2). 

**Table 2.** Screening of Phytochemical Contents of Three Different Extracts of *Leuenbergeria bleo* Leave

Constituent of Detection and Type of Tests	Results ^a^		
	Petroleum Ether	Chloroform	Methanol
**Carbohydrate**			
Molisch test	-	-	-
**Proteins and free amino acids**			
Millon’s test	-	-	-
**Steroids and triterpenoids**			
Libermann Burchard test	+	++	+++ ^b^
Salkowski test	+	++	+++
**Alkaloids**			
Wagner’s reagent	-	+	++
Hager’s reagent	-	+	++
**Tannin and Phenolic compound**			
5% Ferric Chloride solution test	+	+	++
**Flavonoid**			
Shinoda test	+	++	+++

^a^ (+) indicates the presence of secondary metabolites and (-) indicates the absence of secondary metabolites.

^b^ Stronger positive results.

### 4.2. Baseline Blood Glucose Levels in Normal Albino Rats

The baseline fasting blood glucose levels were measured in all experimental groups prior to any treatment. The results, as shown in Table 3, indicated that the glucose levels were consistent across all groups, with no significant differences observed (P > 0.05). The mean glucose levels ranged from 102 - 106 mg/dL, confirming the homogeneity of the animal model at the beginning of the study. These baseline values provided a reliable reference point for subsequent comparisons following the induction of diabetes and treatment with the plant extracts.

**Table 3.** Fasting Blood Glucose Level of Different Groups on Normal Albino Rats (N = 6) ^a, b^

Groups	Blood Glucose Level (mg/dL)
**1**	105.83 ± 2.69
**2**	105.17 ± 2.44
**3**	105.00 ± 1.86
**4**	103.67 ± 1.65
**5**	102.17 ± 0.95

^a^ Values are expressed as mean ± SD.

^b^ Mean values were not significantly different; P > 0.05.

### 4.3. Blood Glucose Levels Following Alloxan-Induced Diabetes

Following the administration of alloxan monohydrate at a dose of 140 mg/kg (30), a significant increase in blood glucose levels was observed in the diabetic-induced group compared with the normal control group (Table 4). The blood glucose levels in the diabetic rats rose sharply within the first 48 hours, increasing by approximately 34% from the initial measurement. This marked elevation in glucose levels confirmed the successful induction of diabetes in the experimental animals.

**Table 4.** Fasting Blood Glucose Levels in Alloxan Monohydrate Induced Diabetic Rats ^a^

Experimental Groups	Blood Glucose Level (mg/dL, h) ^b^			
	1	12	24	48
**Normal control (n = 6) ** ^**c**^	109.67 ± 3.21	108.33 ± 3.11	107.67 ± 2.43	112.00 ± 2.67
**Diabetic induced group (n = 24) ** ^**d**^	251.17 ± 7.42	278.00 ± 4.20	316.67 ± 4.21	383.33 ± 5.27

^a^ Values are expressed as mean ± SD.

^b^ N = 6 for control group and 24 for diabetic group.

^c^ Not significantly different (P > 0.5) compared with normal group before Alloxan monohydrate.

^d^ Significantly different (P < 0.5) compared with normal control group before Alloxan monohydrate.

### 4.4. Effects of Leuenbergeria bleo Extracts on Body Weight in Diabetic Rats

The body weights of the rats were monitored throughout the treatment period to assess the effects of diabetes and the therapeutic potential of the *L. bleo* extracts. As shown in Table 5, the diabetic control group presented a significant reduction in body weight over the 5-day study period, with approximately 26.72% of their initial body weight being lost. In contrast, the groups treated with the chloroform and methanol extracts, as well as the standard metformin group, presented relative stabilization or a slight increase in body weight. The methanol extract group presented a 6.83% increase in body weight, whereas the chloroform extract group presented an 8.07% increase.

**Table 5.** Effect of Extracts of *Leuenbergeria bleo* on Body Weight in Alloxan Monohydrate Induced Diabetic Rats Over a Five-day-Treatment Period (N = 6) ^a^

Experimental Groups	Body Weight		
	Day 1	Day 3	Day 5
**Normal control**	194.50 ± 4.77	193.50 ± 5.21	193.50 ± 4.94
**Diabetic control**	205.83 ± 4.05 ^b, c^	155.67 ± 1.84 ^d, e^	150.83 ± 1.60 ^d, e^
**Standard reference (100 mg/kg/d)**	202.67 ± 3.34 ^b, f^	188.33 ± 3.69 ^b, g^	190.17 ± 3.65 ^b, g^
**Chloroform extract (500 mg/kg/d)**	200.33 ± 4.08 ^b, f, c^	182.00 ± 6.18 ^b, g, c^	184.17 ± 6.31 ^b, g, c^
**Methanol extract (500 mg/kg/d)**	204.83 ± 4.87 ^b, f, c^	185.50 ± 4.40 ^b, g, c^	190.83 ± 5.13 ^b, g, c^

^a^ Values are expressed as mean ± SD.

^b^ Not significantly different compared to the normal group (P > 0.05).

^c^ Not significantly different compared to the standard group (P > 0.05).

^d^ Significantly different compared to the normal group (P < 0.05).

^e^ Significantly different compared to the standard group (P < 0.05).

^f^ Not significantly different compared to the diabetic group (P > 0.05).

^g^ Significantly different compared to the diabetic group (P < 0.05).

### 4.5. Antihyperglycemic Effects of Leuenbergeria bleo Extracts in Diabetic Rats

**Figure 1.**
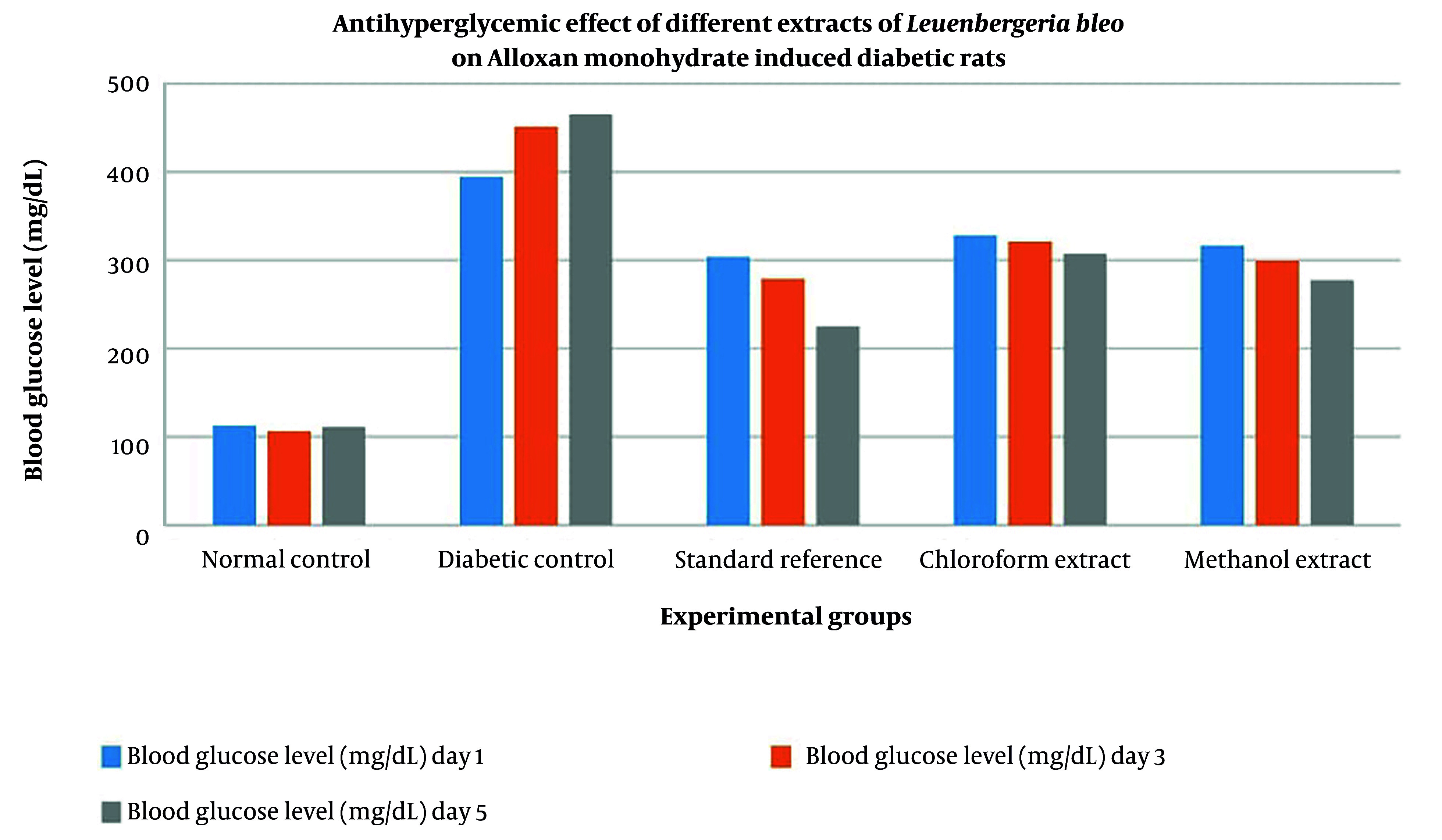
Antihyperglycemic effect of different extracts of *Leuenbergeria bleo* leaves on alloxan monohydrate-induced diabetic rats

The antihyperglycemic effects of the *L. bleo* extracts were evaluated by measuring blood glucose levels at days 1, 3, and 5 of treatment. The results indicate that both the chloroform and methanol extracts significantly reduced blood glucose levels in diabetic rats compared with those in the untreated diabetic control group (Figure 1). The methanol extract, in particular, had a stronger glucose-lowering effect, reducing blood glucose levels by 12.24% by day 5, whereas a 6.36% reduction was observed with the chloroform extract. The standard reference group treated with metformin presented the most pronounced reduction in glucose levels, with a 25.88% decrease by day 5. The methanol extracts reduced glucose levels by 12.24% (Cohen’s d = 2.96, 95% CI: 1.72 - 4.20), while the chloroform extract showed a 6.36% reduction (Cohen’s d = 1.89, 95% CI: 0.91 = 2.87), indicating large to very large effect sizes despite the small sample size.

## 5. Discussion

Diabetes-induced weight loss is primarily due to the catabolic effects of insulin deficiency, leading to muscle wasting and fat breakdown (21, 22). The major finding of this study is that *L. bleo* leaf extracts, particularly the methanol extract, exhibited significant antihyperglycemic effects in alloxan-induced diabetic rats, consistent with their phytochemical profiles. In this study, it was demonstrated that methanol, the most polar solvent, yielded the highest extract amount (5.94%), followed by chloroform (5.60%), which aligns with the presence of bioactive compounds such as flavonoids, alkaloids, tannins, phenolics, and steroids detected in these extracts (Table 2). The presence of flavonoids like quercetin and rutin, known to inhibit carbohydrate-metabolizing enzymes including α-glucosidase, contributes to reducing postprandial glucose levels (23). Alkaloids may further activate the AMPK pathway, enhancing glucose and lipid metabolism (24). Additionally, phenolic acids provide antioxidant properties that help mitigate oxidative stress, a major factor in pancreatic β-cell dysfunction in diabetes (25). These compounds may act synergistically, amplifying the antihyperglycemic effect of *L. bleo* extracts.

The methanol extract demonstrated the most potent glucose-lowering activity, reducing blood glucose by 12.24% by day 5, followed by the chloroform extract with a 6.36% reduction. Both extracts also contributed to stabilizing body weight, counteracting the 26.72% weight loss observed in untreated diabetic controls. Methanol and chloroform extracts produced weight increases of 6.83% and 8.07%, respectively, comparable to the effects seen with metformin treatment (Table 5). These findings suggest that *L. bleo* extracts alleviate diabetic symptoms, likely through improved glycemic control and attenuation of diabetes-associated catabolism.

Acute oral toxicity studies have confirmed the safety of *L. bleo* extracts up to 2500 mg/kg with no adverse effects reported (26). The phytochemical constituents identified, including phytosterols, alkaloids, tannins, phenolics, and flavonoids, have well-documented antidiabetic activities in other plants, supporting their contribution to the observed effects in this study.

Overall, *L. bleo* shows promise as a natural antidiabetic agent that may provide a safer, more cost-effective alternative to synthetic drugs. Future research should focus on elucidating the molecular mechanisms underlying its effects, particularly insulin signaling, glucose metabolism, and oxidative stress pathways. Isolation and characterization of the active compounds are essential to facilitate the development of targeted therapies. Furthermore, long-term studies assessing chronic efficacy, safety, dose-response relationships, and effects on lipid metabolism and insulin sensitivity will provide comprehensive insight into the therapeutic potential of *L. bleo*.

### 5.1. Limitations and Future Directions

This study adopted a five-day treatment protocol to evaluate short-term antihyperglycemic effects. Similar durations have been validated in published models, including Nkono et al. and Omabe et al., which demonstrated significant glucose reductions within this window (27, 28). While suitable for acute-phase assessment, future studies should extend treatment duration to evaluate sustained efficacy and safety. Petroleum ether extract was indeed prepared and subjected to preliminary phytochemical screening. The results indicated minimal presence of bioactive compounds, particularly those relevant to antidiabetic activity. Consequently, it was excluded from in vivo testing to focus on the more pharmacologically promising chloroform and methanol extracts. This rationale has been clarified in the revised methodology section (section 2.2), and the screening results are presented in Table 2. Only a single dose (500 mg/kg) was tested in this study, selected based on prior toxicity data indicating safety up to 2500 mg/kg (26), which aimed to establish preliminary efficacy.

## Data Availability

The dataset presented in the study is available on request from the corresponding author during submission or after publication.
